# Triticeae crop genome biology: an endless frontier

**DOI:** 10.3389/fpls.2023.1222681

**Published:** 2023-07-20

**Authors:** Zhaoxu Gao, Jianxin Bian, Fei Lu, Yuling Jiao, Hang He

**Affiliations:** ^1^ State Key Laboratory of Protein and Plant Gene Research, School of Advanced Agriculture Sciences and School of Life Sciences, Peking University, Beijing, China; ^2^ Peking University Institute of Advanced Agricultural Sciences, Shandong Laboratory of Advanced Agricultural Sciences in Weifang, Shandong, China; ^3^ State Key Laboratory of Plant Cell and Chromosome Engineering, Institute of Genetics and Developmental Biology, The Innovative Academy of Seed Design, Chinese Academy of Sciences, Beijing, China; ^4^ University of Chinese Academy of Sciences, Beijing, China; ^5^ CAS-JIC Centre of Excellence for Plant and Microbial Science (CEPAMS), Institute of Genetics and Developmental Biology, Chinese Academy of Sciences, Beijing, China; ^6^ State Key Laboratory for Protein and Plant Gene Research, School of Life Sciences, Peking University, Beijing, China; ^7^ Peking-Tsinghua Center for Life Sciences, Center for Quantitative Biology, Academy for Advanced Interdisciplinary Studies, Peking University, Beijing, China; ^8^ State Key Laboratory of Plant Genomics, Institute of Genetics and Developmental Biology, The Innovative Academy of Seed Design, Chinese Academy of Sciences, Beijing, China

**Keywords:** Triticeae, wheat, barley, rye, genome sequencing, pan-genome

## Abstract

Triticeae, the wheatgrass tribe, includes several major cereal crops and their wild relatives. Major crops within the Triticeae are wheat, barley and rye which are important for human consumption, animal feed, and rangeland protection. Species within this tribe are known for their large genomes and complex genetic histories. Powered by recent advances in sequencing technology, researchers worldwide have made progress in elucidating the genomes of Triticeae crops. In addition to assemblies of high-quality reference genomes, pan-genome studies have just started to capture the genomic diversities of these species, shedding light on our understanding of the genetic basis of domestication and environmental adaptation of Triticeae crops. In this review, we focus on recent signs of progress in genome sequencing, pan-genome analyses, and resequencing analysis of Triticeae crops. We also propose future research avenues in Triticeae crop genomes, including identifying genome structure variations, the association of genomic regions with desired traits, mining functions of the non-coding area, introgression of high-quality genes from wild Triticeae resources, genome editing, and integration of genomic resources.

## Introduction

### Relationship between Triticeae crop

The tribe Triticeae within the subfamily Pooideae includes 27 genera, and 501 diploid and polyploid species (Soreng et al., 2015). Triticeae comprises several major crop species such as barley (*Hordeum vulgare* L.), rye (*Secale cereale* L.) and wheat, including bread wheat (*Triticum aestivum* L. ssp*. aestivum*) and durum wheat (*Triticum turgidum* L. ssp*. durum*). Wheat and barley are the founder crops of the Neolithic Agricultural Revolution in the Fertile Crescent of the Middle East, and they continue to be major cereal crops of temperate regions globally to the present day. Not surprisingly, extensive efforts have been made to study Triticeae crops throughout the world. However, Triticeae crops lagged since crop studies have entered the molecular genetic era, mainly due to the large genome sizes of Triticeae crops. After the completion of rice (*Oryza sativa*) and maize (*Zea mays*) genome sequencing, it has been relatively routine to identify causal genes underlying agronomic traits (Liu and Yan, 2019).

The bread wheat genome is substantially larger than barley and has a complex evolutionary history. Like barley, bread wheat also originated in the Fertile Crescent. This hexaploid species contains three subgenomes (A, B, and D) obtained through two hybridization events. In the first round of hybridization, two diploid wild ancestors created wild emmer wheat (*T. turgidum* L. ssp. *dicoccoides*) (BBAA) (Haas et al., 2019). The second hybridization event occurred at least 8,800 years ago and involved a cross between free-threshing tetraploid wheat and the donor of the D genome, *Aegilops tauschii* Coss. (DD) (Salamini et al., 2002; Zhao et al., 2023). Furthermore, the D subgenome possibly resulted from an ancient hybridization event followed by gene loss (Marcussen et al., 2014). Following these duplication events, highly similar genes coexist among the B, A, and D subgenomes. The large Triticeae crop genomes have a high content of transposable elements, which impeded genome assembly. Furthermore, wheat is polyploid, further complicating genome sequencing and assembly.

### Development of sequencing technology

**Figure 1:**
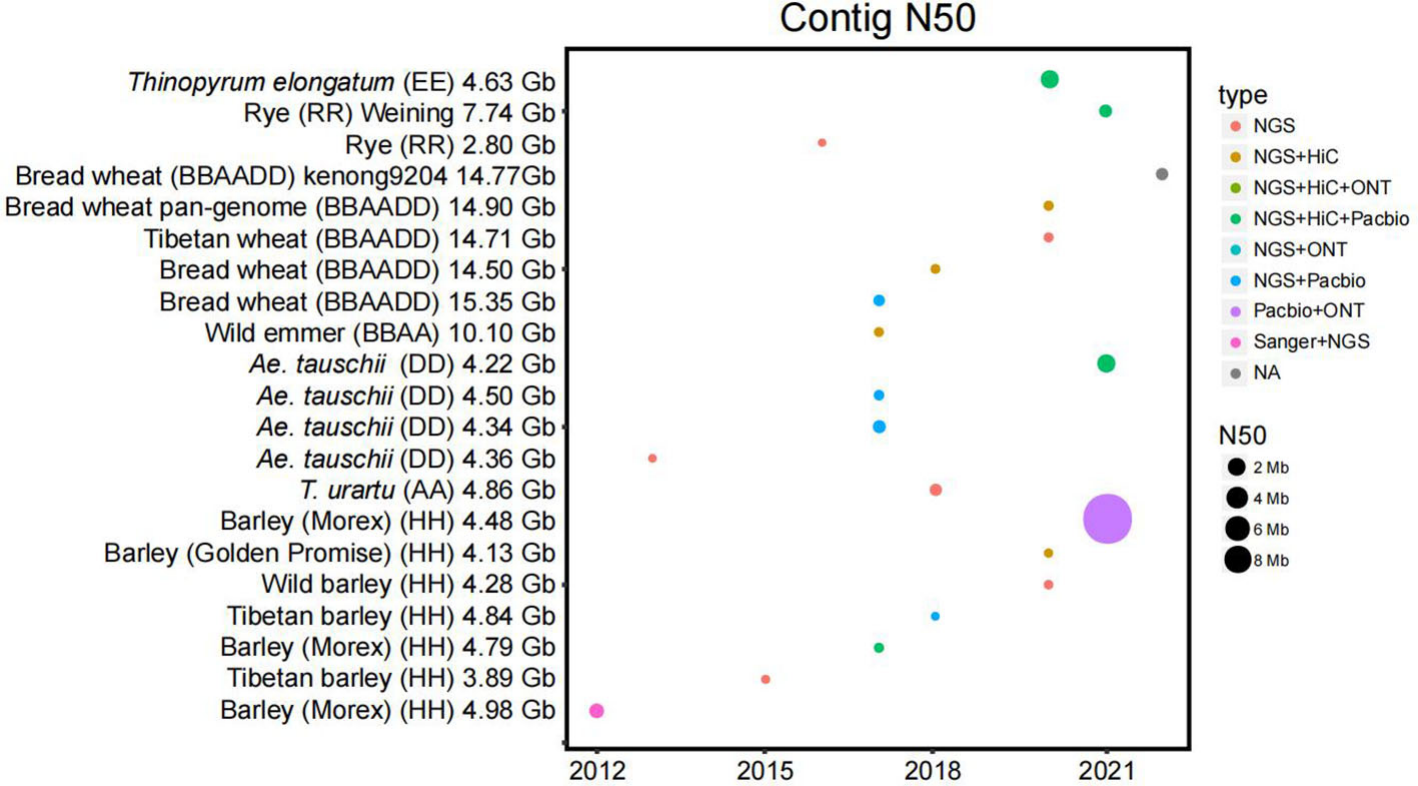
Statistics of the published Triticeae crop genomes. The contig N50 (the sequence length of the shortest contig at 50% of the total assembly size) is plotted by the year of publication. The size of each dot is the numerical value of N50. The sequencing platforms are color-coded. The sequencing technologies and the size of N50 have driven a large improvement over the years.

The genome of Arabidopsis has assembled two decades ago, which heralds the beginning of plant genome sequencing (Sun et al., 2022a). A reference-level genome provides basic information for plant research. Importantly, well-assembled and annotated genomes are even more important in studies of crop domestication, natural variation, and breeding (Sun et al., 2022a). DNA sequencing started with Sanger sequencing in 1977. Shotgun sequencing emerged in 1982. With the advent of ‘next-generation’ DNA sequencing (NGS) in 2005, the number of genome assemblies increased vastly. The third-generation sequencing technology has been developed for nearly a decade (Shendure et al., 2017). With the development of these sequencing technologies, algorithms, and software for high throughput data analysis were also continuously developed and updated. All these technological advances had made significant contributions to complex genome assembling (Shendure et al., 2017) (**Figure 1**). To continuously explore and update the huge genome of wheat and barley, International Wheat Genome Sequencing Consortium (IWGSC) and International Barley Genome Sequencing Consortium (IBGSC) were established. The research on functional genomics of wheat lagged far behind that of rice and maize, which restricted the development of gene cloning and molecular design breeding techniques for important agronomic traits. A high-quality reference genome sequence map is the infrastructure to achieve a breakthrough (Brenchley et al., 2012).

## Triticeae crop genome biology

### Using the development of sequencing technology, barley has obtained an accurate genome

Barley is the trendsetter in Triticeae genome sequencing. Many technological breakthroughs for Triticeae sequencing are first demonstrated in barley (**Table 1**). Barley (2*n* = 2*x* = 14, HH) is an important cereal crop with a genome size of 5.1 Gb and a repeat sequence ratio of up to 84%. The first effort was to develop a chromosome draft sequence based on flow-sorted chromosomes (Mayer et al., 2011). Following that, a whole-genome shotgun-based draft genome sequence was released for ‘Morex’, a hulled barley cultivar, and 26,159 high-confidence genes were annotated (Consortium TIBGS, 2012). By employing chromosome conformation capture (Hi-C), a highly contiguous chromosome-scale genome assembly was obtained, in which the scaffold N50 length was increased to 1.9 Mb from 1.4 kb in the previous draft genome, and 39,734 high-confidence genes were annotated (Mascher et al., 2017). Subsequently, Genebanks was established through the collection of barley data. Genebanks can provide insight into the global population structure of domesticated barley by analyzing genome-wide genotyping data for almost all barley germplasm (Milner et al., 2018).

**Table 1:** Presently available reference genomes for Triticeae crops.

Crop/species	Ploidy	Genome size	Contig N50	Scaffold N50	Year	References
Barley (Morex) (HH)	Diploid	4.98 Gb	904.00 kb		2012	(Consortium TIBGS, 2012)
Tibetan barley (HH)	Diploid	3.89 Gb	18.07 kb	242.00 kb	2015	(Zeng et al., 2015)
Barley (Morex) (HH)	Diploid	4.79 Gb	79.00 kb	1.90 Mb	2017	(Mascher et al., 2017)
Tibetan barley (HH)	Diploid	4.84 Gb	5.94 kb	173.83 kb	2018	(Dai et al., 2018)
Wild barley (HH)	Diploid	4.28 Gb	35.4 kb	724.93 kb	2020	(Liu et al., 2019)
Barley (Golden Promise) (HH)	Diploid	4.13 Gb	22.4 kb	4.14 Mb	2020	(Schreiber et al., 2020)
Barley (Morex V2) (HH)	Diploid	4.65 Gb		40.20 Mb	2019	(Monat et al., 2019)
Barley (Morex) (HH) (5 accessions)	Diploid	4.14 Gb- 4.48 Gb	69.6 Mb- 87.6 Mb	14.20Mb-118.90 Mb	2021	(Mascher et al., 2021)
Barley pan-genome (HH) (20 accessions)	Diploid	3.80 Gb- 4.50 Gb		5.00 Mb- 42.70 Mb	2020	(Jayakodi et al., 2020)
*T. urartu* (AA)	Diploid	4.66 Gb		63.69 kb	2013	(Ling et al., 2013)
*T. urartu* (AA)	Diploid	4.86 Gb	344 kb	3.67 Mb	2018	(Ling et al., 2018)
*Ae. tauschii* (DD)	Diploid	4.36 Gb	4.52 kb	57.60 kb	2013	(Jia et al., 2013)
*Ae. tauschii* (DD)	Diploid	4.22 Gb		31.73 Mb	2017	(Luo et al., 2017)
*Ae. tauschii* (DD)	Diploid	4.34 Gb	486.80 kb	521.70 kb	2017	(Zimin et al., 2017b)
*Ae. tauschii* (DD)	Diploid	4.50 Gb	112.60 kb	12.10 Mb	2017	(Zhao et al., 2017)
*Ae. tauschii* (DD) pan–genome (DD) (4 accessions)	Diploid	4.12 Gb- 4.22 Gb	1.90 Mb-2.20 Mb	48.70 Mb-76.60 Mb	2021	(Zhou et al., 2021)
Wild emmer (BBAA)	Tetraploid	10.10 Gb	57.38 kb	6.96 Mb	2017	(Avni et al., 2017)
Wild emmer (BBAA)	Tetraploid	10.37 Gb		72.63 Mb	2019	(Zhu et al., 2019)
Wild emmer (Zavitan) (BBAA)	Tetraploid	11.10 Gb		1.30 Mb	2019	(Monat et al., 2019)
Durum wheat (BBAA)	Tetraploid	10.45 Gb		6.00 Mb	2019	(Maccaferri et al., 2019)
Bread wheat (Chr 3B)	Hexaploid	995.0 Mb			2008	(Yu et al., 2008)
Bread wheat (BBAADD)	Hexaploid	10.20 Gb			2014	(International Wheat Genome Sequencing Consortium, 2014)
Bread wheat (BBAADD)	Hexaploid	9.10 Gb		24.80 kb	2015	(Chapman et al., 2015)
Bread wheat (BBAADD)	Hexaploid	13.43 Gb		88.80 kb	2017	(Clavijo et al., 2017)
Bread wheat (BBAADD)	Hexaploid	15.35 Gb	232.66 kb		2017	(Zimin et al., 2017a)
Bread wheat (BBAADD)	Hexaploid	14.50 Gb	51.80 kb	7.00 Mb	2018	(Appels et al., 2018)
Tibetan wheat (BBAADD)	Hexaploid	14.71 Gb	66.26 kb	37.62 Mb	2020	(Guo et al., 2020)
Bread wheat (BBAADD)	Hexaploid	15.70 Gb		2.30 Mb	2019	(Monat et al., 2019)
Bread wheat pan-genome (BBAADD) (15 accessions)	Hexaploid	14.10 Gb- 14.90 Gb	16.40 kb- 83.47 kb	68.50 kb- 49.70 Mb	2020	(Walkowiak et al., 2020)
Bread wheat pan-genome (BBAADD) (4 accessions)	Hexaploid	14.53 Gb-14.71 Gb		6.87 Mb-72.09 Mb	2021	(Zhu et al., 2021)
Bread wheat (BBAADD) Fielder	Hexaploid	14.70 Gb		21.00 Mb	2021	(Sato et al., 2021)
Bread wheat (BBAADD) kenong9204	Hexaploid	14.77Gb	366 kb	21.87 Mb	2022	(Shi et al., 2022)
Bread wheat (BBAADD) AK58	Hexaploid	14.75Gb			2021	(Jizeng Jia et al., 2021)
Rye (RR)	Diploid	2.80 Gb	1.71 kb	9.45 kb	2016	(Bauer et al., 2017)
Rye (RR) Weining	Diploid	7.74 Gb	480.35 kb	1.04 Gb	2021	(Li et al., 2021)
Rye (RR) Lo7	Diploid	6.74 Gb		29.40 Mb	2021	(Rabanus-Wallace et al., 2021)
*Thinopyrum elongatum* (EE)	Diploid	4.63 Gb	2.15 Mb	73.24 Mb	2020	(Wang et al., 2020a)

The tool TRITEX (Illumina PE/MP, 10 × Genomics and Hi-C technology) was used to obtain a 4.65 Gb barley reference genome (Morex V2), with increased scaffold N50 up to 40.2 Mb, and 32,787 high confidence genes were annotated. The BUSCO evaluation index was increased to 97.8% from 92.5% in the Morex V1 version (Monat et al., 2019). Based on the TRITEX strategy, the Morex V3 barley reference genome with a size of 4.50 Gb was assembled by combining third-generation of sequencing techniques (PacBio long read length and cyclic consensus sequencing data; Nanopore long read length data). The scaffold N50 jumped to 118.9 Mb and 35,827 high-confidence genes were annotated (Mascher et al., 2021).

### Genomic assembly of the Tibetan hulless barley and wild barley

The Tibetan hulless barley (*Hordeum vulgare* L. var. *nudum*), also called “Qingke” in Chinese, is an important staple food for Tibetans (Zeng et al., 2015). The genome of Tibetan hulless barley “Lhasa Goumang” was assembled using the whole-genome shotgun method. The assembly obtained a 3.89-Gb size genome, accounting for 87% of the Tibetan hulless barley genome, and containing 39,197 protein-coding genes (Zeng et al., 2015). With the development of the latest third-generation sequencing technology, 4.84G genome assembly of another hulless barley cv. Zangqing320 has been completed (Dai et al., 2018).

As the ancestor of cultivated barley, wild barley (*Hordeum spontaneum*) has important research and application value in the evolution and domestication fields of barley, as well as genetic breeding. Therefore, it is of great significance to study the beneficial genes related to disease resistance and stress tolerance in wild barley. A 4.28 Gb high-quality draft assembly of wild barley accession AWCS276 from Iran was obtained with in-depth sequencing using the whole genome shotgun sequencing (150×), accounting for 93% of the entire genome. A total of 36,395 coding protein genes were predicted using genomic and transcriptome data (Liu et al., 2019). The most effective barley genome for genetic transformation obtained a 4.13-Gb genome by adding Hi-C data to increase the contiguity to full chromosome size (Schreiber et al., 2020). As a wild species of barley and wheat, *Hordeum marinum* Huds. (2n=2x=14, XaXa) had excellent characteristics such as salt tolerance and resistance to water-logging, and enriched genetic resources for crop improvement. A 3816 Mb reference level genome was assembled using third-generation, second-generation, and Hi-C data, with a contig N50 6.83 Mb (Kuang et al., 2022). The genome information and efficient gene editing system of sea barley had important value for the breeding improvement of Triticeae crops.

### Long-sequence sequencing technology propels the progress of the wheat genome

Following barley, the wheat genomes are unmasked step by step (**Table 1**). The hexaploid bread wheat genome is by far the most conspicuous in the Triticeae tribe. The Chinese landrace Chinese Spring (CS) is chosen for sequencing as the reference first. Due to the extremely complex genome of wheat (2*n* = 6*x* = 42 chromosomes), the physical map of the 3B chromosome was first constructed using bacterial artificial chromosome (BAC) (Yu et al., 2008). Six years later, the first reference sequence of the 3B chromosome was finished, providing a proof-of-concept and template for sequencing the remaining chromosomes of wheat (Choulet et al., 2014). The first draft sequence used ~60,000 Illumina-based genic sequences to assemble and putatively assign to subgenomes using *Triticum monococcum*, *Ae. tauschii*, and *Aegilops speltoides* sequence data, a total of 17Gb sequences were assembled, and 94,000-96,000 genes were predicted (Brenchley et al., 2012). Following that, an ordered draft sequence was produced by shotgun sequencing of isolated chromosome arms (International Wheat Genome Sequencing Consortium, 2014). In this version, 124,201 gene loci were identified across homoeologous chromosomes and subgenomes. Subsequently, the assembly length of the wheat genome gradually increased (Chapman et al., 2015; Clavijo et al., 2017; Zimin et al., 2017a). Although high sequence similarity and structural conservation are generally retained between bread wheat subgenomes and corresponding diploid and tetraploid wheat relatives, dynamic and sporadic gene gain, loss, and duplication are also evident. By combining chromosome shotgun sequencing, pseudomolecules with high-density genetic (POPSEQ), BAC-based physical mapping, Hi-C, and Bionano optical mapping, a substantially updated full reference genome release, IWGSC RefSeq v1.0, was obtained. In this version, 97% (14.1 Gb) of the assembled 14.5-Gb genome (N50 of super scaffold = 22.8 Mb) was assigned and ordered to 21 chromosomes (Appels et al., 2018). The classical BAC-by-BAC strategy provides highly ordered, high-confidence, chromosome-level sequences. This approach has been applied to the long arm of chromosome 7D (Feng et al., 2020). In the more recently updated IWGSC RefSeq v2.1 version, using optical maps and PacBio long reads, scaffolds were anchored and corrected, increasing the length of pseudomolecules by 168 Mb (Zhu et al., 2021). The 14.26 Gb genome of the French bread wheat cultivar Renan was assembled by long reads produced on the Oxford Nanopore Technology PromethION device, with a scaffold N50 of 48 Mb (Aury et al., 2022). A 14.7-Gb assembled chromosome of South African bread wheat Kariega was obtained by combining high-fidelity long reading, optical mapping, and chromosome conformation capture (Athiyannan et al., 2022). The resulting assembly sequence has an order of magnitude more continuity than previous wheat sequences.

### The genomic research of AABB, AA, DD, other subgenomes, and wild and semi-wild wheat is conducted to accelerate the genomic research of wheat

To decipher the complex wheat genomes, parallel efforts have been made to sequence and assemble diploid wheat ancestors, including *Triticum urartu* (AA) and *Ae. tauschii* (DD), tetraploid wild emmer and durum wheat (BBAA), and hexaploid bread wheat (BBAADD) (**Table 1**). As for barley, these efforts have been accompanied by rapid progress in sequencing technologies and assembly algorithms. As such, reference genome assemblies have been substantially improved over just a few years.

For example, the first released draft of the *T. urartu* (AA) genome assembly employed an Illumina platform-based whole-genome shotgun sequencing strategy to obtain a total of 4.66 Gb genome sequence with a scaffold N50 length of 63.69 kb (Ling et al., 2013). By combining bacterial artificial chromosome (BAC)-by-BAC sequencing and PacBio long read-based whole-genome shotgun sequencing, the *T. urartu* genome assembly was subsequently improved, and a genome of 4.86 Gb in size with a scaffold N50 length of 344 kb was obtained (Ling et al., 2018). Similar progress has been made in *Ae. tauschii* (DD) genome sequencing (Jia et al., 2013; Luo et al., 2017; Zhao et al., 2017; Zimin et al., 2017b; Gaurav et al., 2021; Zhou et al., 2021), and emmer and durum wheat genome sequencing (Avni et al., 2017; Maccaferri et al., 2019). Through the optical maps, the genome sequence of wild emmer wheat was improved (Zhu et al., 2019).

### Genomic assembly of the other varieties of hexaploid wheat

The high-quality reference genome of Tibetan semi-wild wheat was obtained with 14.71 Gb in size and 118,078 annotated genes. It is the second reference genome of hexaploid wheat published in the world (Guo et al., 2020). Using PacBio circular consensus sequencing (CCS) with the HiFi approach, the most easily transformed wheat ‘Fielder’ genome was obtained, with an N50 greater than 20 Mb (Sato et al., 2021). By combining the sequencing strategy including DeNovoMAGIC-2, PacBio sequencing, and multiple mapping techniques, a high-quality genome of nitrogen-efficient wheat variety “Kenong 9204” was assembled, with a 14.77 Gb genome size, and the N50 was 21.87 Mb (Shi et al., 2022). AK58 was an elite Chinese common wheat cultivar and had a draft genome at the chromosome level (Jizeng Jia et al., 2021). The N50 of assembling was 715 Mb. A draft genome of Tibetan semi-wild wheat Zang1817 (*Triticum aestivum* ssp. *tibetanum Shao*) was assembled with an N50 of 37.62 Mb (Guo et al., 2020).

### The rye genome made a significant breakthrough in 2021

By employing these technical advances, the recently finished rye (*Secale cereale* L.) genome assemblies have reached a high standard (**Table 1**). Rye is a diploid crop grown extensively as a grain, cover crop, and forage crop especially on marginal land. Rye is exceptionally stress tolerant and has been extensively introgressed into bread wheat to improve grain yield and biotic and abiotic stress tolerance (Crespo-Herrera et al., 2017; Ayalew et al., 2018). Through shotgun sequencing of the whole genome of the winter rye inbred line Lo7, a 2.8-Gb whole-genome draft sequence of rye was obtained (Bauer et al., 2017). This reference sequence represents almost the entire low-copy portion of the rye genome. Rye is an out-crossing crop, and there are more exchanges with wild gene pools. The diploid 7.9 Gb rye genome was much larger than the syntenic diploid barley and bread wheat subgenomes (Li et al., 2021; Rabanus-Wallace et al., 2021). The two released genome assemblies were of high quality, with a scaffold N50 size of 1.04 Gb for the Weining rye genome and 29.44 Mb for the Lo7 rye genome. These high-quality rye genome assemblies reveal a recent long terminal repeat-retrotransposon burst around 0.5 million years ago, explaining the genome size expansion. Notably, a series of genes related to disease resistance, as well as genes involved in seed storage, heading data, frost tolerance, and fertility restoration, have been identified in the rye chromosome arm 1RS. This chromosome arm has been of broad interest as it has translocated to bread wheat chromosome 1BL to form 1B1R translocation lines, which show strong resistance to stripe rust diseases and powdery mildew (Crespo-Herrera et al., 2017).

### Pan-genome analysis in Triticeae crop

The release of reference genomes further enabled pan-genome analyses of Triticeae crops. Triticeae cereals are widely cultivated and selected in divergent environments throughout the world. Pan-genome analyses take advantage of the rich genetic resources and century-long germplasm collection efforts for these crops. The initial efforts relied on microarrays that can detect single nucleotide polymorphism (SNP) (Winfield et al., 2012). Following that, Illumina platform-based short reads resequencing had been employed, which did not rely on prior knowledge of the genome polymorphism (Montenegro et al., 2017). However, short reads resequencing cannot identify genome structure variations, and the application of long reads-based resequencing and *de novo* assembly has further expanded our understanding of genome diversity (Hao et al., 2020; Jayakodi et al., 2020; Walkowiak et al., 2020). In addition to the technical evolution, the sizes of the population being analyzed have also expanded fast (**Table 2**).

**Table 2:** Population genomic diversity studies in barley and wheat.

Crop/species	SNPs	Other variations	Population size	Genotyping method	References
Barley	1,688,807	143,872 indels	267	Exome capture	(Russell et al., 2016)
Barley	544,318		433	Assay	(Pankin et al., 2018)
Barley		69,192,653 SNVs and indels	13	GBS	(Wang et al., 2018)
Barley	171,263		22,626	GBS	(Milner et al., 2018)
Barley	19,248,055		196	WGS	(Zeng et al., 2020)
Barley	57,238,496	3,379,287indels	33	WGS	(Bian et al., 2020)
Barley		1,586,262 PAVs	20	*De novo* assembly	(Jayakodi et al., 2020)
Tibetan barley	36,469,491	2,281,198 indels	10	WGS	(Zeng et al., 2015)
Tibetan barley	56,349,359	3,913,456 indels	437	WGS and exome capture	(Ling et al., 2018)
*Ae. tauschii*	25,927,240	1,592,193 indels	278	WGS	(Zhou et al., 2021)
Wild emmer	46301637		47	WGS	(Wang et al., 2020b)
Tetraploid wheat	17,340		1,856	Wheat 90K array	(Maccaferri et al., 2019)
*T. urartu*, *Ae. speltoides*, *Ae. tauschii*	A: 1,271; B: 1,218; D: 2,203			GSP	(Akhunov et al., 2010)
Hexaploid wheat	511,439			Array	(Winfield et al., 2012)
Hexaploid and tetraploid wheat	46,977		37	Wheat 90K array	(Wang et al., 2014)
Hexaploid wheat	1,556,654	161,719 indels	62	Exome capture and GBS	(Jordan et al., 2015)
Di-, tetra- and hexaploid wheat	541,466	54,473 indels	487	Exome capture	(Pont et al., 2019)
Hexaploid wheat		27,933 DArTseq markers; 312 831 SNP markers	1002	DArTseq and wheat 660K array	(Zhou et al., 2018)
Hexaploid wheat	78,606		44,624	GBS	(Zhu et al., 2019)
Hexaploid wheat	7.3M		890	Exome capture	(He et al., 2019)
Di-, tetra- and hexaploid wheat	84,594,991	11,628,085 indels; 105,316 deletions; 100,509 duplications	183	WGS	(Cheng et al., 2019)
Hexaploid wheat	4,009		2,657	Wheat 90K array	(Fradgley et al., 2019)
Hexaploid wheat	186,646	114,538 SilicoDArT presence/absence	79,191	Wheat 90K array	(Sansaloni et al., 2020)
Hexaploid wheat	46,431,479		308	WGS	(Guo et al., 2020)
Hexaploid wheat	60,922,016	2,540,666	145	WGS	(Hao et al., 2020)
Di-, tetra- and hexaploid wheat	103,899,092		414	WGS	(Zhou et al., 2020)
bread wheat and its relatives	77,734,258		795	WGS	(Zhao et al., 2023)
Di-, tetra- and hexaploid wheat	1,609,246		649	Wheat 660K array	(Wang et al., 2021b)
Hexaploid wheat	99,991,379		149		(Joynson et al., 2021)
Egyptian emmer wheat	99,078		64	WGS	(Scott et al., 2019)
Hexaploid wheat		162,541 PAV genes; 65,850 CNV genes	11	*De novo* assembly	(Walkowiak et al., 2020)
Rye		8,626,622 SNVs	11	WGS	(Bauer et al., 2017)

SNP, single nucleotide polymorphism; SNV, single nucleotide variant; DArTSeq, Diversity Arrays Technology with sequencing; PAV, presence-absence variation; CNV, copy number variation; RQA, reference-quality pseudomolecule assembly; WGS, whole genome sequencing; GBS, genotyping-by-sequencing.

Low-resolution pan-genome analyses uncovered the genetic adaption, domestication, and breeding history of Triticeae crops. Several recent pan-genome studies have been carried out on wheat (Montenegro et al., 2017; Walkowiak et al., 2020) and barley (Jayakodi et al., 2020). A year before the publication of Chinese Spring RefSeq genome v1.0, the first pan-genome containing 18 wheat species was constructed through highly in-depth next-generation sequencing. However, 12,150 genes with variants were not found in Chinese Spring. These genes are associated with stress and defense (Montenegro et al., 2017). Another study reported ten reference-quality pseudomolecule assemblies and five scaffold-level assemblies of hexaploid wheat. These high-quality assemblies facilitated the identification of extensive structural rearrangements and introgressions from wild relatives, which are related to grain yield and quality, resistance to stresses, and adaptation to diverse environments (Walkowiak et al., 2020). Improved long-read sequencing-based assembly held promise to identify large genome structure variations (Jayakodi et al., 2020; Walkowiak et al., 2020). In barley, a study using 20 representative accessions from 19,778 worldwide domesticated varieties not only resolved barley breeding history but also provided detailed views of two frequently identified inversions in current elite barley germplasm (Jayakodi et al., 2020).

### Evolutionary genomics analysis in Triticeae crop

Before the whole genome resequencing of wheat was conducted, 500,000 SNPs were obtained through targeted re-sequencing based upon NimbleGen array technology, of which 80% belonged to A, B, and D subgenome variations and 20% belonged to inter variability among varieties (Winfield et al., 2012). Through the whole exome capture and genotyping-by-sequencing approaches, the first haplotype map of wheat was generated, detailing the genetic differences among wheat sample lines worldwide (Jordan et al., 2015). Approximately 4500 years ago, wheat was introduced to China. SNP markers reveal the genetic diversity of wheat varieties in China (Akhunov et al., 2010; Wang et al., 2014). Meanwhile, using wheat Wheat660K gene chip and DArT-seq technology, the possible transmission routes of wheat after its introduction into China from abroad were analyzed (Zhou et al., 2018). The endemic Tibetan semi-wild wheat in China had been acclimated to Tibet varieties (Guo et al., 2020).

**Figure 2:**
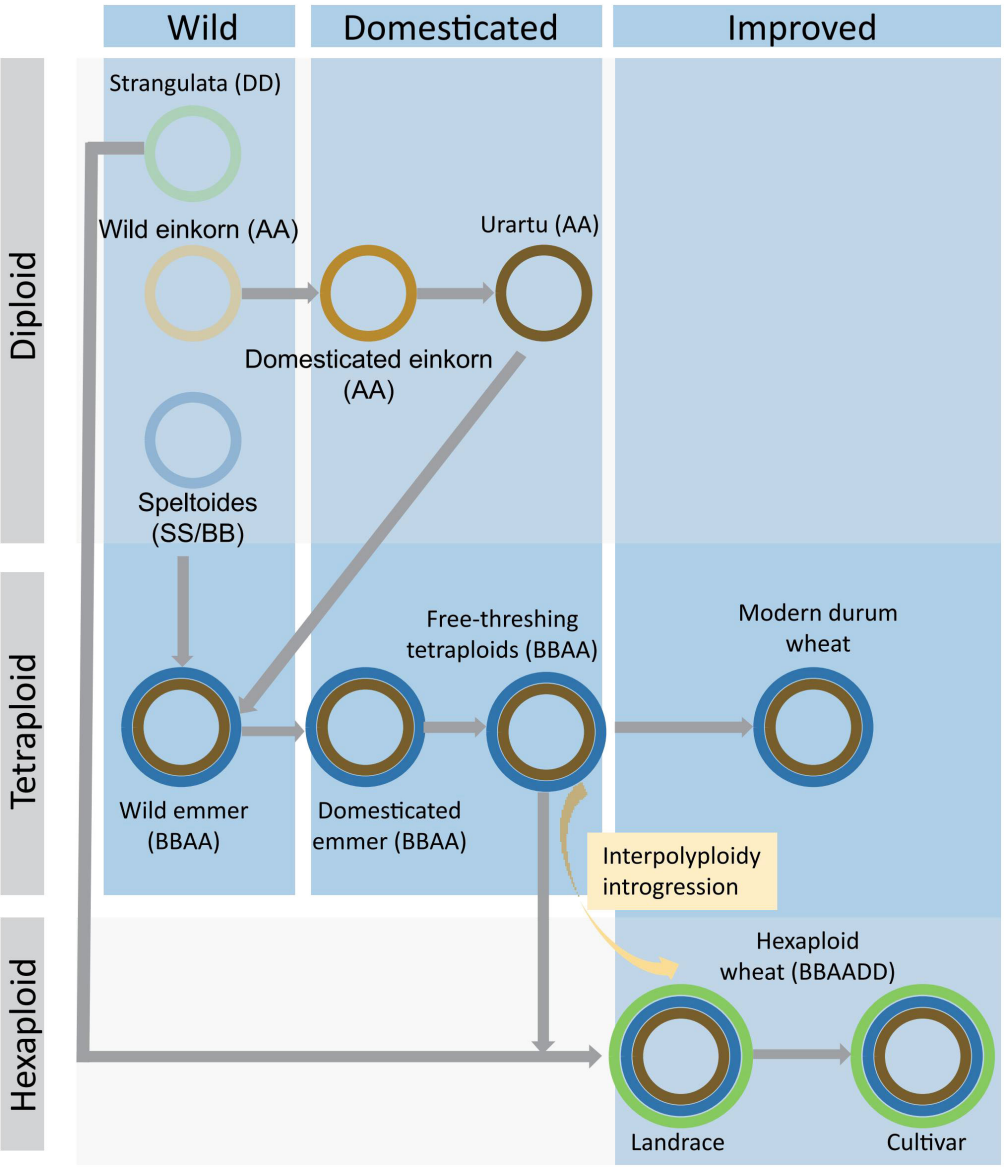
Schematic illustration of the domestication of polyploid wheat. Modern hexaploidy wheat is generated through two polyploidization events from diploid ancestors. Diploid AA is derived from wild einkorn and becomes *urartu* AA through domesticated einkorn. BB, as a maternal parent, hybridizes with AA, which is a paternal parent, to form a tetraploid of BBAA. Modern durum arose through domesticated emmer and free-threshing tetraploids wheat. The hybridization between BBAA and DD resulted in the final hexaploid wheat. During the process from landrace to cultivar, the interpolyploidy introgression of tetraploids from BBAA can increase the diversity of hexaploid wheat. The blue rectangle represents the domestication processes, while the gray rectangle shows different ploidy.

Although different landraces and cultivars were selected by different research teams, several key common conclusions can be drawn from bread wheat resequencing studies (Balfourier et al., 2019; Hao et al., 2020; Sansaloni et al., 2020; Zhou et al., 2020; Wang et al., 2021b; Zhao et al., 2023). It has long been proposed that after its formation in the Fertile Crescent during the Neolithic period, bread wheat germplasm spread via two routes along with ancient human migration. Genome sequence analyses provided solid evidence for this hypothesis, as population structure separated European and New World varieties from Asian varieties (Balfourier et al., 2019). After their dissemination, bread wheat populations adapt to local environments to become landraces. A notable finding was that Indian dwarf wheat (*T. aestivum* ssp. *sphaerococcum*), endemic to southern Pakistan and northwestern India, was likely a geographically isolated and low-diversity taxon (Zhou et al., 2020). A genome-wide genetic variation map of wheat (VMap 1.0) was constructed by resequencing 414 wheat materials (Zhou et al., 2020). Subsequently, this map was upgraded using whole genome sequencing data from 795 wheat materials (VMap 1.1) (Zhao et al., 2023). A large fraction of genome variations present in landraces had been lost in cultivars (Sansaloni et al., 2020). In the process of origin and domestication of polyploid wheat, interploidy introgression played an important role in restoring population genetic diversity (Wang et al., 2022). These works have revealed the original mechanism of domesticated wheat and the evolution law of long-term gradual domestication, accelerating the analysis of wheat evolution (Zhou et al., 2020; Wang et al., 2022; Zhao et al., 2023) (**Figure 2**). Nevertheless, there have also been reshufflings over time, presumably related to modern breeding programs. For example, modern Chinese landraces possessed significant contributions from European varieties (Balfourier et al., 2019; Hao et al., 2020; Zhou et al., 2020). Through the resequencing of 145 landmark cultivars of wheat, the process of genome remodeling and optimization of wheat cultivars in China in the past 70 years has been revealed. It has been found that there were significant breeding selection asymmetries among the three subgenomes of wheat, providing a scientific basis and ideas for cultivating candidate backbone parents and assembly breeding (Hao et al., 2020). More variations from landraces had been incorporated into cultivars released after the 1960s (Balfourier et al., 2019). In the 1970s, wheat had the highest diversity of varieties in China (Wang et al., 2021b).

For the study of barley domestication trajectory, people sequenced the genome of the 6,000-year-old barley, and genetic analysis supports the current hypothesis that barley domestication occurred in the Upper Jordan Valley (Mascher et al., 2016; Russell et al., 2016). Targeted resequencing analysis showed that although the domesticated barley originated from the Fertile Crescent, the candidate domestication genes originated in its eastern and western regions (Pankin et al., 2018). Qingke was introduced to southern Tibet most likely via north Pakistan, India, and Nepal between 4,500 and 3,500 years ago. And Qingke maybe not be the origin of barley of the low genetic diversities (Zeng et al., 2018).

By whole genome sequencing and analyzing the 116 varieties containing wild, weedy, and cultivated rye germplasm resources, it was revealed that cultivated rye was domesticated from weedy rye (Sun et al., 2022b).

Alien introgressions have been identified by further including pan-genome analyses of tetraploid and diploid ears of wheat (**Figure 2**; **Table 2**) (Zhou et al., 2020). Tetraploid wheat is the primary contributor to the high genetic diversity of the bread wheat A and B subgenomes (Cheng et al., 2019; He et al., 2019; Zhou et al., 2020). Among them, free-threshing domesticated emmers contribute more diversity than wild emmers or other domesticated emmers (Zhou et al., 2020). By contrast, diploid *T. urartu* had made very limited introgression contributions to bread wheat, which was distinct from previous assumptions (Zhou et al., 2020). Notably, gene flow from the tetraploid emmers significantly contributed to the genetic diversity of the A and B subgenomes in bread wheat. By contrast, the barrier to gene flow from the diploid *T. urartu* or *Ae. tauschii* to bread wheat was much higher, as they cannot cross easily with bread wheat. Gene flow from wild wheat (*T. turgium* subsp. *dicoccoides*) had a significant impact on single nucleotide polymorphisms (SNPs) throughout the wheat genome (He et al., 2019; Pont et al., 2019). After the publication of the wheat genome (Appels et al., 2018), the study by Cheng et al. was the first one to analyze genetic variations using resequencing data, and it was found that the genetic diversity of hexaploid wheat originated from intraspecific and interspecific hybridization (Cheng et al., 2019). Gene flow (gene migration) and chromosome rearrangement had important contributions to the formation of genetic diversity in common wheat, and the impact of gene flow on future breeding strategies had been evaluated (Przewieslik-Allen et al., 2021). The whole genome sequencing of 3000-year-old Egyptian emmer wheat revealed genetic similarities between ancient Egyptian wheat and modern Arab and Indian wheat, indicating the early eastward and southward spread of ancient Egyptian wheat (Scott et al., 2019). Using chips and genome resequencing, population genetic analysis was conducted on 168 wild emmer wheat. It was found that the wild emmer wheat utilized different adaptive strategies in the same domain environment, resulting in different evolutionary pathways (Wang et al., 2020b). Similarly, strong genetic selection occurred in wild barley populations for soil drought adaptation (Wang et al., 2018) and soil ecological differences (Bian et al., 2020).

Large genome size and genetic complexity significantly hamper gene function analyses in Triticeae crops through map-based cloning of key regulatory genes. Pan-genome analyses provided an efficient alternative strategy for identifying genes under selection during domestication and breeding (Haas et al., 2019). New inference models and methods have been formulated for identifying selective sweeps and localizing selection target sites throughout the genome. These methods aim at uncovering beneficial alleles from the vast majority of neutral variations. In barley, pan-genome studies had identified genomic regions putatively under selection, which harbored regulatory genes underlying several morphological traits (Milner et al., 2018; Jayakodi et al., 2020). Bristling awns is a beneficial trait for seed dispersal in wild barley but a nuisance for farmers when harvesting and for animals when chewing (Elbaum et al., 2007). Regulatory genes for awn length and awn roughness were found in GWAS peaks (Milner et al., 2018). Pan-genome analyses of hexaploid and diploid wheat populations had identified regions that harbor genes encoding putative regulators of agronomic traits (Walkowiak et al., 2020; Zhou et al., 2021). For example, analysis of the brittle rachis trait revealed convergent adaptation in different wheat populations (Zhou et al., 2020). Domesticated emmer wheat and einkorn wheat harbor independent mutations in orthologs of the barley *Btr1* gene, which controls seed shattering.

### Identification of selected genes in Triticeae crop

When phenotypic data is available, a genome-wide association study (GWAS) is possible, in which genomes from a population are scanned for genetic variations that can be used to predict phenotypic traits (Liu and Yan, 2019). Using genomic tools to improve wheat is crucial for accelerating the improvement of trait inheritance (Russell et al., 2016; Juliana et al., 2019; Pont et al., 2019; Guo et al., 2020; Zeng et al., 2020; Joynson et al., 2021). It has been reported that 35 key traits had genomic predictability and demonstrated the potential of genomic selection for wheat. A large genome-wide association study is also conducted to identify several important loci for trait associations in 50 traits in South Asia, Africa, and the Americas (Juliana et al., 2019). In an exon sequencing analysis in wheat, 48 and 40 genomic loci were identified that they were significantly related to flowering stage and plant height variation, including known genes that control flowering stage (*Ppd*, *VRN*, *FDL*, *WPCL*) and *Rht* genes that affect plant height (Pont et al., 2019). Targeted sequencing data combined with genome-wide association analysis reveal genetic loci related to photosynthesis in wheat (Joynson et al., 2021). In Tibetan semi-wild wheat, important de-domestication sites were identified through association analysis, providing further evidence for de-domestication (Guo et al., 2020). Through exon sequencing analysis and a common garden experiment involving 267 landraces and wild accessions collected from diverse geographical locations, a study in barley shed light on environmental adaption, and identified allelic variation in barley associated with geographical adaptation, such as heading days related to seasonal temperature and plant height related to dryness variables (Russell et al., 2016). Using a metabolite-based genome-wide association study (mGWAS), the metabolic gene base for adapting to high levels of UV-B in highland barley was revealed, providing a reference for the improvement of highland barley varieties and the study of molecular genetic mechanisms of biological and abiotic stresses in other crops (Zeng et al., 2020). In addition, complete genomic information can facilitate the cloning of key genes. For example, after obtaining a reference-quality genome assembly from *Aegilops sharonensis*, the stem rust resistance gene *Sr62* was cloned (Yu et al., 2022). The search for these key genes in development and resistance contributes to the development of Triticeae crop breeding.

### The development of the Triticeae crop genome has greatly promoted the progress of Triticeae crop epigenome analysis

The method of mutation accumulation has been used to prove that epigenetic modifications and environmental changes can affect the rate and location of mutations in the genome, and the importance of epigenetic modifications in mutation rate variation has been demonstrated (Habig et al., 2021) (**Table 3**). Before the release of the IWGSC RefSeq v1.0 genome, the first epigenome map of wheat was drawn, and the results showed that the methylation of the three subgenomes and diploid wheat ancestors *Aegilops tauschii* is highly conserved (Gardiner et al., 2015). With the improvement of genomic information, more and more epigenetic studies have been conducted (Geng et al., 2019; Li et al., 2019; Concia et al., 2020; Yuan et al., 2020; Habig et al., 2021; Jia et al., 2021; Liu et al., 2021; Wang et al., 2021a). Genomic methylation can affect genome-specific gene expression and specific biological processes, such as affecting plant disease resistance (Geng et al., 2019; Li et al., 2019). Genome merging and segregation in tetraploid to hexaploid wheat cause dynamic and reversible DNA methylation, and these changes were related to changes in gene expression and transposon activity (Yuan et al., 2020). The histone modification of H3K27me3 is relatively stable in different ploidy wheat, but the peak levels of H3K27me2 increased with increasing ploidy levels (Liu et al., 2021). The subgenomic differentiation activity of homologous regulatory elements was related to the dynamic regulation of methylase complexes, demethylases, and H3K27me3 (Wang et al., 2021a).

**Table 3:** Epigenomic studies in barley and wheat.

Crop/species	Type of epigenome	Genome version	Year	References
Hexaploid wheat	DNA methylation	(Brenchley et al., 2012)	2015	(Gardiner et al., 2015)
Diploid progenitor *Aegilops tauschii* accession AL8/78	DNA methylation	Ensemble_IWGSP2.25	2019	(Geng et al., 2019)
Bread wheat	DNA methylation	IWGSC reference sequence (version 1.0)	2019	(Li et al., 2019)
Tetraploid wheat (ETW, AABB)	DNA methylation	SNP-corrected Chinese Spring genome sequence (WGAv1)	2020	(Yuan et al., 2020)
Natural hexaploid bread wheat (NHW, *T. aestivum* L. cv Canthach, AABBDD)	DNA methylation	SNP-corrected Chinese Spring genome sequence (WGAv1)	2020	(Yuan et al., 2020)
Natural tetraploid wheat (NTW, *T. turgidum* L. subsp. *durum*, AABB)	DNA methylation	corresponding SNP-corrected genome sequences	2020	(Yuan et al., 2020)
*Ae. tauschii* subsp. *strangulata* (line RL5288, DD)	DNA methylation	corresponding SNP-corrected genome sequences	2020	(Yuan et al., 2020)
Resynthesized hexaploid wheat (RHW, AABBDD)	DNA methylation	SNP-corrected Chinese Spring genome sequence (WGAv1)	2020	(Yuan et al., 2020)
*Triticum urartu* (AA, accession TMU06)	Histone H3K27me2 and H3K27me3	A subgenome of *Triticum aestivum* cv. Chinese Spring (IWGSC RefSeq v1.0)	2021	(Liu et al., 2021)
*T. turgidum* ssp. *dicoccoides* (wAABB, line TD265)	Histone H3K27me2 and H3K27me3	AB subgenome of *Triticum aestivum* cv. Chinese Spring (IWGSC RefSeq v1.0)	2021	(Liu et al., 2021)
*T. turgidum* ssp. *durum* (dAABB)	Histone H3K27me2 and H3K27me3	AB subgenome of *Triticum aestivum* cv. Chinese Spring (IWGSC RefSeq v1.0)	2021	(Liu et al., 2021)
*T. aestivum* cv. Chinese Spring (AABBDD)	Histone H3K27me2 and H3K27me3	*Triticum aestivum* cv. Chinese Spring (IWGSC RefSeq v1.0)	2021	(Liu et al., 2021)
*Ae. tauschii* ssp. *strangulata* (DD, line TQ18)	Histone H3K27me2 and H3K27me3	D subgenome of *Triticum aestivum* cv. Chinese Spring (IWGSC RefSeq v1.0)	2021	(Liu et al., 2021)
*Triticum aestivum* cultivar “Chinese Spring”	H3K27me3	IWGSC RefSeq genome assembly (version 1.0)	2021	(Wang et al., 2021a)
*Triticum aestivum* cultivar “Chinese Spring” and the rapeseed cultivar Westar	Hi-C	IWGSC RefSeq genome assembly (version 1.0)	2020	(Concia et al., 2020)
*Triticum aestivum* cultivar AK58	Hi-C	AK58 genome	2021	(Jia et al., 2021)
*Triticum aestivum* cultivar “Chinese Spring”	OCEAN-C	IWGSC RefSeq genome assembly (version 1.0)	2022	(Yuan et al., 2022)
natural allotetraploid wheat (*T. turgidum* L. subsp. *durum*, AABB)	OCEAN-C	AB subgenome of IWGSC RefSeq genome assembly (version 1.0)	2022	(Yuan et al., 2022)
*Aegilops tauschii* subsp. *strangulata* (line RL5288, DD)	OCEAN-C	D subgenome of IWGSC RefSeq genome assembly (version 1.0)	2022	(Yuan et al., 2022)

OCEAN-C, The open chromatin enrichment, and network Hi-C.

The physical chromosome organization is vital in the polyploid genome (Concia et al., 2020; Jia et al., 2021; Yuan et al., 2022). Transposons play an important role in stabilizing subgenomic stability (Jia et al., 2021), which influences transcriptional regulation in wheat (Concia et al., 2020). Recent research has used OCEAN-C technology to map open chromatin interactions in different ploidy wheat, integrating chromatin accessibility, histone modification, and transcriptome to deeply analyze the long-distance interactions of open elements during the polyploidy process in hexaploidy wheat (Yuan et al., 2022).

## Prospects

Sequencing technology has advanced rapidly in the past decade, inspiring the development of genome mapping, assembly methods, and population genetic algorithms (Mardis, 2017; Shendure et al., 2017; Sun et al., 2022a). For example, the recently developed fast and accurate long-read sequencing by circular consensus sequencing technology holds promise to assemble highly repetitive Triticeae genomes, as the recent pilot experiment in barley suggested (Mascher et al., 2021). In the Triticeae genome, accurate assembly of huge tandem repeats, complex chromosome rearrangements, and translocation regions are extensively existing, which may prevent precise genome assembly. In existing technologies, the assembly results can be corrected through the assistance of Hi-C data. In addition, advanced molecular cytogenetic approaches called the Oligo-FISH painting system, may provide future experimental instead of computational correction or verification of the complex Triticeae assembly (Li et al., 2020). This new technology can accurately identify non-homologous chromosomes in polyploid Triticeae crops.

How to fully exploit the identified genome variations is another challenge. Graphic-pangenomics has been an emerging method to identify regions under selection and introgression. Annotation of the genome structure variations, including coding regions and non-coding (regulatory) regions, is also a key breakthrough to expect. When applied to pan-genome studies, long reads-based resequencing and *de novo* assembly would identify genome structure variations. Although already applied in barley and wheat, the broader application is an urgent need to identify more large-scale variations, which often affect agronomic traits (Montenegro et al., 2017; Jayakodi et al., 2020). To systematically mine regions contributing to agronomic traits, pan-genome sequences need to be combined with high-throughput phenotypic data to accelerate breeding programs.

The exploration and utilization of key genes play an important role in promoting the genetic improvement of wheat yield. For example, the “Green Revolution” in the middle of the last century significantly reduced the plant height of wheat and achieved an increase in yield by using dwarf genes such as *Rht-B1b* or *Rht-D1b*. Similar to the development in rice (*Oryza sativa*) and maize (*Zea mays*), it has been beneficial to identify causal genes underlying agronomic traits based on complete genome reference (Liu and Yan, 2019). *TaCol-B5* was found by map-based cloning, which had functions in modifying spike architecture and enhancing grain yield in wheat (Zhang et al., 2022). Based on accurate genomic information, differences in amino acid sites result in changes in gene function. A large fragment of approximately 500 Kb was missing on the short arm of chromosome 4B, leading to the loss of three closely linked genes. This site is a semi-dwarf site that synergistically enhances wheat yield and nitrogen utilization efficiency (Song et al., 2023). The comprehensive genomic information provides a foundation for the search of these genes, and the base or sequence changes in their regulatory mechanisms also depend on the accuracy of genomic information.

In addition, wheat breeding has long benefited from distant hybridization with wild relatives. Crossing with wild Triticeae species, such as *Aegilops* spp., *Thinopyrum* spp., *Elytrigia elongata syn. Lophopyrum elongatum*, and *Hordeum* spp., commonly introduce chromosome fragments into bread wheat to provide enhanced stress tolerance and/or disease resistance. For example, a series of Xiaoyan varieties, derived from the cross between bread wheat and *Thinopyrum ponticum*, has a high-stress tolerance and enhanced photosynthesis and had been broadly cultivated in China. Sequencing these wild species would also be a frontier in Triticeae genome biology. Recently, sequencing *Thinopyrum elongatum* has helped the identification of a Fusarium head blight-resistant gene that was introduced from this wild relative into bread wheat through distant hybridization breeding (Wang et al., 2020a). Genome sequencing would accelerate comparable studies to better utilize the rich genetic resource embedded in the entire Triticeae tribe.

Genomic selection (GS) is a breeding strategy of predicting and choosing the superior populations through the construction of statistical models based on the molecular markers and phenotypes of the training population (Meuwissen et al., 2001). Furthermore, the research on GS has received widespread attention and significant progress in maize and rice (Cui et al., 2019; Xiao et al., 2021). However, due to the limitations of the large and complex genome in wheat, there is relatively little progresses in this area. Recently, in wheat, it was found that plant height marker, DTHD and DTMT marker, and disease resistance marker can achieve average prediction efficiency at 74%, 76%, and 85% levels, respectively (Juliana et al., 2019). These results indicate that a small number of molecular markers can be used for GS breeding in wheat, and excellent prediction results can be achieved. These also provide directions for future GS breeding research in wheat. Firstly, due to the limited genetic variation in hexaploid wheat, fewer molecular markers can represent most variations, thus effectively predicting the agronomic traits of hexaploid wheat. Currently, this severely restricted the further improvement of wheat traits. So, expanding the GS genetic variation population, including tetraploid and distant hybridizations, is an effective approach. Secondly, the current variations in the wheat populations were got from GBS (Genotyping-by-Sequencing) method in the wheat population. It will more effectively improve the prediction efficiency of the model by capturing and modeling many variations in the wheat population, as well as structural variation. The ongoing development of third-generation sequencing combined with next-generation sequencing can assist in this process. Lastly, due to wheat being a widely cultivated crop worldwide, environmental plasticity is one of the key traits in GS, which is also an important direction for the wheat improvement of environmental adaptability. At present, there is relatively little research that integrates environmental factors into the GS models, which will also be an important research direction for applying the GS model to the production of higher yield and stronger stress resistance wheat varieties.

## Author contributions

HH and YJ designed the scope of the manuscript. ZG, JB, FL, YJ, and HH together wrote the manuscript. ZG, YJ, and HH prepared the figures. All authors contributed to the article and approved the submitted version.
