# Corrigendum: Triticeae crop genome biology: an endless frontier

**DOI:** 10.3389/fpls.2023.1280660

**Published:** 2023-10-06

**Authors:** Zhaoxu Gao, Jianxin Bian, Fei Lu, Yuling Jiao, Hang He

**Affiliations:** ^1^ State Key Laboratory of Protein and Plant Gene Research, School of Advanced Agriculture Sciences and School of Life Sciences, Peking University, Beijing, China; ^2^ Peking University Institute of Advanced Agricultural Sciences, Shandong Laboratory of Advanced Agricultural Sciences in Weifang, Shandong, China; ^3^ State Key Laboratory of Plant Cell and Chromosome Engineering, Institute of Genetics and Developmental Biology, The Innovative Academy of Seed Design, Chinese Academy of Sciences, Beijing, China; ^4^ University of Chinese Academy of Sciences, Beijing, China; ^5^ CAS-JIC Centre of Excellence for Plant and Microbial Science (CEPAMS), Institute of Genetics and Developmental Biology, Chinese Academy of Sciences, Beijing, China; ^6^ State Key Laboratory for Protein and Plant Gene Research, School of Life Sciences, Peking University, Beijing, China; ^7^ Peking-Tsinghua Center for Life Sciences, Center for Quantitative Biology, Academy for Advanced Interdisciplinary Studies, Peking University, Beijing, China; ^8^ State Key Laboratory of Plant Genomics, Institute of Genetics and Developmental Biology, The Innovative Academy of Seed Design, Chinese Academy of Sciences, Beijing, China

**Keywords:** Triticeae, wheat, barley, rye, genome sequencing, pan-genome

In the published review, there was an error in the classification of oat. Due to the differences in inflorescence between oat and tribe Triticeae, oat cannot be included in tribe Triticeae. Given their strict classification, we have removed the description of oat section in our review to make the content of this review more rigorous.

A correction has been made to **Abstract,** paragraph one, page 1.

The sentence previously stated:

“Major crops within the Triticeae are wheat, barley, rye, and oat, which are important for human consumption, animal feed, and rangeland protection.”

The corrected sentence appears below:

“Major crops within the Triticeae are wheat, barley and rye which are important for human consumption, animal feed, and rangeland protection.”

A correction has been made to the **Keywords,** page 1.

The keywords previously stated:

“Triticeae, wheat, barley, rye, oat, genome sequencing, pan-genome”

The corrected keywords appear below:

“Triticeae, wheat, barley, rye, genome sequencing, pan-genome”

A correction has been made to **Introduction, *Relationship between Triticeae crop*,** paragraph one, page 2.

The sentence previously stated:

“Triticeae comprises several major crop species such as barley (*Hordeum vulgare* L.), rye (*Secale cereale* L.), oat (*Avena sativa* L.), and wheat, including bread wheat (*Triticum aestivum* L. ssp. aestivum) and durum wheat (*Triticum turgidum* L. ssp. durum).”

The corrected sentence appears below:

“Triticeae comprises several major crop species such as barley (*Hordeum vulgare* L.), rye (*Secale cereale* L.) and wheat, including bread wheat (*Triticum aestivum* L. ssp. *aestivum*) and durum wheat (*Triticum turgidum* L. ssp. *durum*).”

A correction has been made to **Triticeae crop genome biology** section, ***The oat genome made a significant breakthrough in 2022*,** page 6.

The section has been removed.

A correction has been made to **Table 1**, pages 3 – 4.

**Table 1:** Presently available reference genomes for Triticeae crops.

Crop/species	Ploidy	Genome size	Contig N50	Scaffold N50	Year	References
Barley (Morex) (HH)	Diploid	4.98 Gb	904.00 kb		2012	(Consortium TIBGS, 2012)
Tibetan barley (HH)	Diploid	3.89 Gb	18.07 kb	242.00 kb	2015	(Zeng et al., 2015)
Barley (Morex) (HH)	Diploid	4.79 Gb	79.00 kb	1.90 Mb	2017	(Mascher et al., 2017)
Tibetan barley (HH)	Diploid	4.84 Gb	5.94 kb	173.83 kb	2018	(Dai et al., 2018)
Wild barley (HH)	Diploid	4.28 Gb	35.4 kb	724.93 kb	2020	(Liu et al., 2019)
Barley (Golden Promise) (HH)	Diploid	4.13 Gb	22.4 kb	4.14 Mb	2020	(Schreiber et al., 2020)
Barley (Morex V2) (HH)	Diploid	4.65 Gb		40.20 Mb	2019	(Monat et al., 2019)
Barley (Morex) (HH) (5 accessions)	Diploid	4.14 Gb- 4.48 Gb	69.6 Mb- 87.6 Mb	14.20Mb-118.90 Mb	2021	(Mascher et al., 2021)
Barley pan-genome (HH) (20 accessions)	Diploid	3.80 Gb- 4.50 Gb		5.00 Mb- 42.70 Mb	2020	(Jayakodi et al., 2020)
*T. urartu* (AA)	Diploid	4.66 Gb		63.69 kb	2013	(Ling et al., 2013)
*T. urartu* (AA)	Diploid	4.86 Gb	344 kb	3.67 Mb	2018	(Ling et al., 2018)
*Ae. tauschii* (DD)	Diploid	4.36 Gb	4.52 kb	57.60 kb	2013	(Jia et al., 2013)
*Ae. tauschii* (DD)	Diploid	4.22 Gb		31.73 Mb	2017	(Luo et al., 2017)
*Ae. tauschii* (DD)	Diploid	4.34 Gb	486.80 kb	521.70 kb	2017	(Zimin et al., 2017b)
*Ae. tauschii* (DD)	Diploid	4.50 Gb	112.60 kb	12.10 Mb	2017	(Zhao et al., 2017)
*Ae. tauschii* (DD) pan–genome (DD) (4 accessions)	Diploid	4.12 Gb- 4.22 Gb	1.90 Mb-2.20 Mb	48.70 Mb-76.60 Mb	2021	(Zhou et al., 2021)
Wild emmer (BBAA)	Tetraploid	10.10 Gb	57.38 kb	6.96 Mb	2017	(Avni et al., 2017)
Wild emmer (BBAA)	Tetraploid	10.37 Gb		72.63 Mb	2019	(Zhu et al., 2019)
Wild emmer (Zavitan) (BBAA)	Tetraploid	11.10 Gb		1.30 Mb	2019	(Monat et al., 2019)
Durum wheat (BBAA)	Tetraploid	10.45 Gb		6.00 Mb	2019	(Maccaferri et al., 2019)
Bread wheat (Chr 3B)	Hexaploid	995.0 Mb			2008	(Yu et al., 2008)
Bread wheat (BBAADD)	Hexaploid	10.20 Gb			2014	(International Wheat Genome Sequencing Consortium, 2014)
Bread wheat (BBAADD)	Hexaploid	9.10 Gb		24.80 kb	2015	(Chapman et al., 2015)
Bread wheat (BBAADD)	Hexaploid	13.43 Gb		88.80 kb	2017	(Clavijo et al., 2017)
Bread wheat (BBAADD)	Hexaploid	15.35 Gb	232.66 kb		2017	(Zimin et al., 2017a)
Bread wheat (BBAADD)	Hexaploid	14.50 Gb	51.80 kb	7.00 Mb	2018	(Appels et al., 2018)
Tibetan wheat (BBAADD)	Hexaploid	14.71 Gb	66.26 kb	37.62 Mb	2020	(Guo et al., 2020)
Bread wheat (BBAADD)	Hexaploid	15.70 Gb		2.30 Mb	2019	(Monat et al., 2019)
Bread wheat pan-genome (BBAADD) (15 accessions)	Hexaploid	14.10 Gb- 14.90 Gb	16.40 kb- 83.47 kb	68.50 kb- 49.70 Mb	2020	(Walkowiak et al., 2020)
Bread wheat pan-genome (BBAADD) (4 accessions)	Hexaploid	14.53 Gb-14.71 Gb		6.87 Mb-72.09 Mb	2021	(Zhu et al., 2021)
Bread wheat (BBAADD) Fielder	Hexaploid	14.70 Gb		21.00 Mb	2021	(Sato et al., 2021)
Bread wheat (BBAADD) kenong9204	Hexaploid	14.77Gb	366 kb	21.87 Mb	2022	(Shi et al., 2022)
Bread wheat (BBAADD) AK58	Hexaploid	14.75Gb			2021	(Jizeng Jia et al., 2021)
Rye (RR)	Diploid	2.80 Gb	1.71 kb	9.45 kb	2016	(Bauer et al., 2017)
Rye (RR) Weining	Diploid	7.74 Gb	480.35 kb	1.04 Gb	2021	(Li et al., 2021)
Rye (RR) Lo7	Diploid	6.74 Gb		29.40 Mb	2021	(Rabanus-Wallace et al., 2021)
*Thinopyrum elongatum* (EE)	Diploid	4.63 Gb	2.15 Mb	73.24 Mb	2020	(Wang et al., 2020a)

The corrected **Table 1** is below:

A correction has been made to **Figure 1**, page 3.

**Figure 1:**
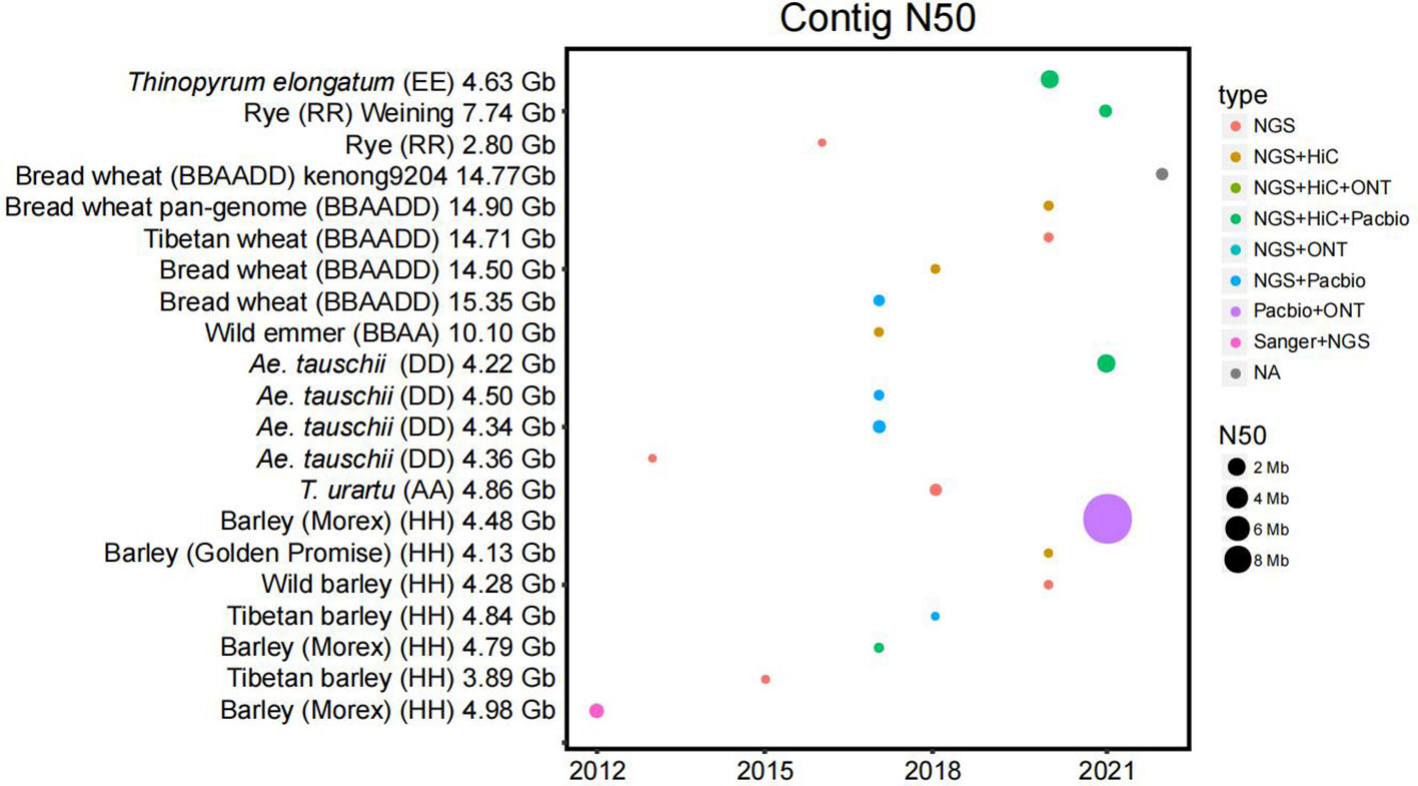
Statistics of the published Triticeae crop genomes. The contig N50 (the sequence length of the shortest contig at 50% of the total assembly size) is plotted by the year of publication. The size of each dot is the numerical value of N50. The sequencing platforms are color-coded. The sequencing technologies and the size of N50 have driven a large improvement over the years.

The corrected **Figure 1** is below:

The authors apologize for this error and state that this does not change the scientific conclusions of the article in any way. The original article has been updated.

